# A Text Book of the Practice of Equine Medicine

**Published:** 1883-10

**Authors:** 


### REVIEWS.

A Text Book of the Practice of Equine Medicine. By William Robertson, F.R.C.V.S., etc. London, Baillière, Tindall and Cox; New York, W. R. Jenkins. 1883.

Students and practitioners of veterinary medicine will hail with satisfaction the appearance of this important work, filling, as it does, a long felt want, namely, a practical, systematic, as well as scientific, and clearly written treatise on the Diseases of the Horse.

Of the eight hundred pages of which the book is composed, thirty are devoted to the consideration of the Theory of Medicine and the remainder to the Practice. This latter section is arranged in two divisions; in the one are described General and Constitutional Diseases, and in the other Local Diseases.

The chapter on Glanders, extending over fifty pages, is remarkably well written. Of its cause the author says: "For my own part, reasoning from analogy and the results of a comparatively limited experience, I have ever regarded the spontaneous origin of glanders in horses as very problematical.

"If capable of being originated *de novo* it is unlike the particular class, the specific communicable diseases to which it belongs, a class feature of which is that they are only capable of propagation by the reception in the healthy of the specific virus manufactured or proceeding from the diseased. Every case of glanders with which I have come in contact has been most reasonably accounted for on the theory of infection or inoculation; in the majority they have been easily and directly traceable to the source."

In the treatment he insists especially on the importance of sanitary measures, thus: "When we consider the fatal nature of this disease and its acknowledged contagiousness in every form of its development, the great pecuniary loss entailed upon the community by its existence and distribution, not to mention the more serious danger to human life, the importance of well-considered and energetically carried out preventive and suppressive measures becomes obvious to all but the most thoughtless and ignorant. Until, however, veterinary medicine is duly recognized and takes its proper place in that conservable section of 'social science,' public health, we can scarcely expect that executive measures sufficiently comprehensive to satisfy even our present knowledge will be adopted and carried out."

The chapters on Influenza, Anthrax and Intestinal diseases are equally worthy of attention.

We hope to see the book very soon in the hands of every veterinarian in this country and highly appreciated as it truly deserves.

Mr. WILLIAM R. JENKINS, of New York, who has just returned from Europe, concluded, while there, arrangements for the publication in America of a number of important veterinary works just issued or shortly to be published. Among these works are Prof. Robertson's "Practice of Equine Medicine," and George Fleming's "Operative Veterinary Surgery," both large and important works, whose authors stand in the very front rank of the veterinary profession. He will also republish here a number of pamphlets on veterinary subjects at the uniform price of 25 cents; which will discuss topics of particular interest to veterinarians, sanitarians, and the medical profession generally.

Arrangements were also made for new editions of Prof. William's standard works on *Veterinary Medicine* and *Veterinary Surgery* on such terms that the American price hereafter will be $7.50 per volume instead of $10.00.

